# Safety and efficacy of unilateral and bilateral pedicle screw fixation for lumbar degenerative diseases by transforaminal lumbar interbody fusion: An updated systematic review and meta-analysis

**DOI:** 10.3389/fneur.2022.998173

**Published:** 2022-10-10

**Authors:** Rui Zhong, Xiali Xue, Runsheng Wang, Jing Dan, Chuanen Wang, Daode Liu

**Affiliations:** ^1^Department of Orthopedics, Affiliated Sports Hospital of Chengdu Sport University, Chengdu, China; ^2^School of Sports Medicine and Health, Chengdu Sport University, Chengdu, China; ^3^Department of Orthopedics, The Third Affiliated Hospital of Guangxi Traditional Chinese Medicine University, Liuzhou, China

**Keywords:** unilateral, bilateral, lumbar degenerative diseases, pedicle screw, transforaminal lumbar interbody fusion

## Abstract

**Background:**

The purpose of this study was to compare the safety and efficacy of unilateral vs. bilateral pedicle screw fixation (BPSF) for lumbar degenerative diseases.

**Methods:**

Electronic databases including PubMed, Web of science, the Cochrane Library, Scopus, MEDLINE, EMBASE, EBSCO were searched by computer. The deadline was set for June 1, 2022. This study included all high-quality randomized controlled trials (RCTs), prospective clinical controlled studies (PRO), and retrospective studies (Retro) that compared unilateral and bilateral pedicle screw fixation in the treatment of lumbar degenerative diseases. Revman5.3 software was used for meta-analysis after two researchers independently screened the literature, extracted data, and assessed the risk of bias in the study.

**Results:**

Fourteen studies with a total of 1,086 patients were included. Compared with BPSF, unilateral pedicle screw fixation (UPSF) has shorter operation time and hospital time, and less blood loss and operation cost, operation time [SMD = −1.75, 95% CI (−2.46 to −1.03), *P* < 0.00001], hospital time [SMD = −1.10, 95% CI (−1.97 to −0.22), *P* = 0.01], Blood loss [SMD = −1.62, 95% CI (−2.42 to −0.82), *P* < 0.0001], operation cost [SMD = −14.03, 95% CI (−20.08 to −7.98), *P* < 0.00001], the ODI after bilateral pedicle screw fixation was lower, and the degree of lumbar dysfunction was lighter, [SMD = 0.19, 95% CI (0.05–0.33), *P* = 0.007], better fusion effect, fusion rate [RR=0.95, 95% CI (0.91–1.00), *P* = 0.04]. VAS-Low back pain [SMD = 0.07, 95% CI (−0.07–0.20), *P* = 0.35], VAS-Leg pain [SMD = 0.18, 95% CI (−0.00–0.36), *P* = 0.05], SF-36 [SMD = 0.00, 95% CI (−0.30–0.30), *P* = 1.00], complications rate [RR = 0.94, 95% CI (0.9154–1.63), *P* = 0.82], the overall difference was not statistically significant.

**Conclusions:**

Currently limited evidence suggests that UPSF significantly reduces blood loss, significantly shortens the operative time and hospital stay, and reduces blood loss and costs. After BPSF, the ODI was lower, the degree of lumbar spine dysfunction was lower, and the fusion rate was significantly higher. The VAS, SF-36, and complications scores of the two groups were comparable, and there was no significant clinical difference.

## Introduction

As the world's population ages, the prevalence of lumbar degenerative diseases (LDD) rises year after year. LDD refers to a group of diseases caused by structural lumbar spine degeneration, which includes lumbar spinal stenosis, degenerative lumbar spondylolisthesis, lumbar disc herniation, and degenerative lumbar scoliosis. At the same time, it is one of the most common diseases and causes of disability that plague the elderly (1). Clinically, patients select various treatment methods based on their symptoms and onset time. Conservative treatment should be considered first for those with mild symptoms and less obvious signs. If conservative treatment is ineffective and normal work or life is jeopardized, surgery is required (2, 3). The main objectives of lumbar fusion surgery include obtaining a solid spinal segmental fusion while restoring the load of the anterior structure and improving the height of the intervertebral disc (4). At present, more and more elderly patients with LDD choose lumbar fusion surgery to alleviate symptoms and improve their quality of life.

Transforaminal lumbar interbody fusion (TLIF) has been evolving since it was first proposed by Albee and Hibbs in 1911 (5). TLIF is regarded as the “gold standard” for treating lumbar degenerative diseases (6), and it is now a recognized surgical technique. Pedicle screw fixation technology, as a traditional surgical method for the treatment of LDD, can improve initial stability, correct deformities, maintain intervertebral height without external fixation, promote intervertebral fusion, accelerate early walking recovery after surgery, and improve fusion rate (7). Furthermore, TLIF has several advantages, including the preservation of interspinal ligaments, minimal dural sac retraction, and less nerve damage, while exposing the lateral intervertebral space and reducing nerve traction (6, 8). Traditional TLIF achieves immediate postoperative stability through bilateral pedicle screw fixation to achieve solid lumbar interbody fusion (BPSF). The stiffness and stability achieved by BPSF posterior fixation of the interbody cage is a valuable surgical strategy that has gained widespread acceptance (9). However, studies in recent years have shown that unilateral pedicle screw fixation (UPSF) can achieve similar results while reducing implant costs, blood loss, and operation time (10). As a result, the absolute necessity of TLIF for bilateral pedicle fixation remains debatable.

We discovered that UPSF treatment of LDD can reduce operation time, thus reducing bleeding, hospital stay, and implantation costs by reviewing previous studies. However, because of the imbalance of fixation provided by UPSF, its stability may be inferior to that of BPSF, and there may be cage subsidence after the operation, which may increase postoperative sagittal imbalance, intervertebral foramen stenosis, and adjacent segment degeneration, and there are still some conflicting results in terms of complications and clinical results (11, 12). BPSF is used to treat lumbar fusion because it can provide initial stability, correct deformities, maintain intervertebral height, promote intervertebral fusion, and speed up patients' postoperative recovery. However, as the number of implants increases, patients will require more extensive dissection, more blood loss, a longer operation time, a higher cost, and possibly more implantation-related complications (13, 14). Some studies also found no difference in clinical efficacy between UPSF and BPSF combined with TLIF in the treatment of single-segment LDD. Unilateral fixation shortens the operation, reduces bleeding, and lowers hospitalization costs (15). It is unclear whether UPSF intervertebral fusion is superior to BPSF. It is still debatable whether to use UPSF or BPSF. Previous meta-analysis studies compared the clinical effects of UPSF and BPSF on LDD. UPSF and BPSF had comparable clinical outcomes, according to the findings (16). However, there were issues with the previous meta-analysis, such as small sample size and poor quality of some literature. Furthermore, new prospective and randomized controlled trials with long-term follow-up have been published in recent years, involving new evidence. We conducted this meta-analysis to clarify these ambiguous findings.

The purpose of this meta-analysis is to evaluate the safety and effectiveness of transforaminal interbody fusion UPSF and BPSF for LDD, and to evaluate the advantages and disadvantages of these two surgical treatments for LDD, to provide the evidence-based medical basis for clinical treatment of LDD.

## Methods

This study was conducted following the guidelines of the Cochrane handbook (17) and was performed by the Preferred Reporting Items for Systematic Reviews and Meta-Analyses (PRISMA) statement (18) (see Supplementary material 1).

### Search strategy

PubMed, web of science, the Cochrane Library, Scopus, MEDLINE, EMBASE, EBSCO and other databases were searched by computer, and all high-quality randomized controlled trials (RCTs), prospective clinical controlled studies (PRO), and retrospective studies (Retro) comparing UPSF and BPSF in the treatment of LDD were searched. The retrieval time limit was from the establishment of the database to June 1, 2022. The following search terms were used: lumbar degenerative diseases, lumbar spinal fusion, transforaminal lumbar interbody fusion (TLIF), pedicle screw fixation, unilateral, and bilateral. These keywords were used as MeSH headings and free text words. We restricted the language to English. In addition, to maximize the search for relevant articles, further articles were obtained by reviewing the references of the selected articles.

### Selection of studies

The retrieved studies will be imported into Endnote X9 to remove duplicates. Two reviewers (ZR and XXL) will independently screen the titles and abstracts according to the pre-established inclusion and exclusion criteria. After that, the full text will be screened as a second filtration. Two researchers will crosscheck the included studies, when consensus could not be reached, a third reviewer (WRS) was consulted to resolve the disagreement.

### Inclusion and exclusion criteria

This study used the PICOS (Participants, Intervention, Comparator, Outcome, and Study design) model to select studies for this review. The inclusion criteria and exclusion criteria are shown in Table 1.

**Table 1:** Inclusion and exclusion criteria.

**Inclusion criterion**	
Participants	Patients with LDD, Age: 18–85 years, symptomatic back and/or leg pain lasting for more than 3 months, and grade 1–3 single-segment degenerative spondylosis or spondylolisthesis
Intervention	Unilateral pedicle screw fixation
Comparator	Bilateral pedicle screw fixation
Outcomes	VAS, ODI, SF-36, JOA, Operation time, Blood loss, Hospital time, Complications rate, Fusion rate. Fusion was defined as the presence of a continuous fusion mass inside or outside the cage on CT imaging. Status was regarded as non-union when the fusion mass on CT was discontinuous
Study design	RCT or PRO or Retro trials
Study language	English language
**Exclusion criterion**	
Have other spinal diseases	Spinal deformities, such as scoliosis and kyphosis;spinal fractures, infection, or tumor
Outcome indicators and data	Lack of outcome indicator data
Completeness of the study report	Duplicate studies, studies reporting too little information, and studies with incomplete data
Types of literature	Review, meeting abstracts, letters, etc

### Data extraction

Data were independently extracted by two reviewers (DJ and WCN), with further discussion with another independent reviewer (LDD). The following data were extracted from each included study: name of the first author, year of publication, study characteristics (sample size and follow-up time), and patients' characteristics (mean age, sex, and spinal level), treatments (type of intervention details), and clinical outcomes: VAS, ODI, SF-36, JOA, operation time, blood loss, hospital time, complications rate, and fusion rate. If the original data was unclear or lacking, the corresponding author was contacted to obtain further information.

### Assessment of study quality

This study used the Physiotherapy Evidence Database (PEDro) tool (http://www.pedro.fhs.usyd.edu.au/scale_item.html) to assess the methodological quality of individual RCTs (19). According to whether the research clearly meets this standard, the project is scored with yes or no (1 or 0). The total score PEDro is obtained by adding the scores of items 2–11, and the total score is between 0 and 10. The higher the score, the better the quality of the method. Studies with PEDro scores ranging from 9 to 10 were considered methodologically to be of “excellent” quality. Studies with PEDro scores ranging from 6 to 8 were considered to be of “good” quality, while studies scoring between 4 and 5 were of “fair” quality. Studies that scored below 4 were of “poor” quality. In this study, we considered a study awarded ≥6 points on the PEDro scale a high-quality study. The independent evaluation of each study was completed by two raters. If there is any difference, it will be decided through discussion or arbitration from the third researcher.

### Data synthesis

All data were statistically analyzed by Revman 5.3 software. Continuous variables were analyzed with the standardized mean difference (SMD) and 95% confidence interval (CI), and risk ratio (RR) was used for pooled analysis for dichotomous variables. Statistical heterogeneity between studies was assessed using *P*- and *I*^2^-values, with *P* < 0.1 and *I*^2^ > 50% showing high heterogeneity, using a random-effects model. When the level of heterogeneity was not significant, a fixed-effects model was used. If heterogeneity is high, subgroup analysis or meta-regression will be performed to explore sources of heterogeneity. Publication bias was assessed using funnel plots. The meta-analysis was set at *P* < 0.05 for the significance level.

## Results

### Study characteristics and qualities

**Figure 1:**
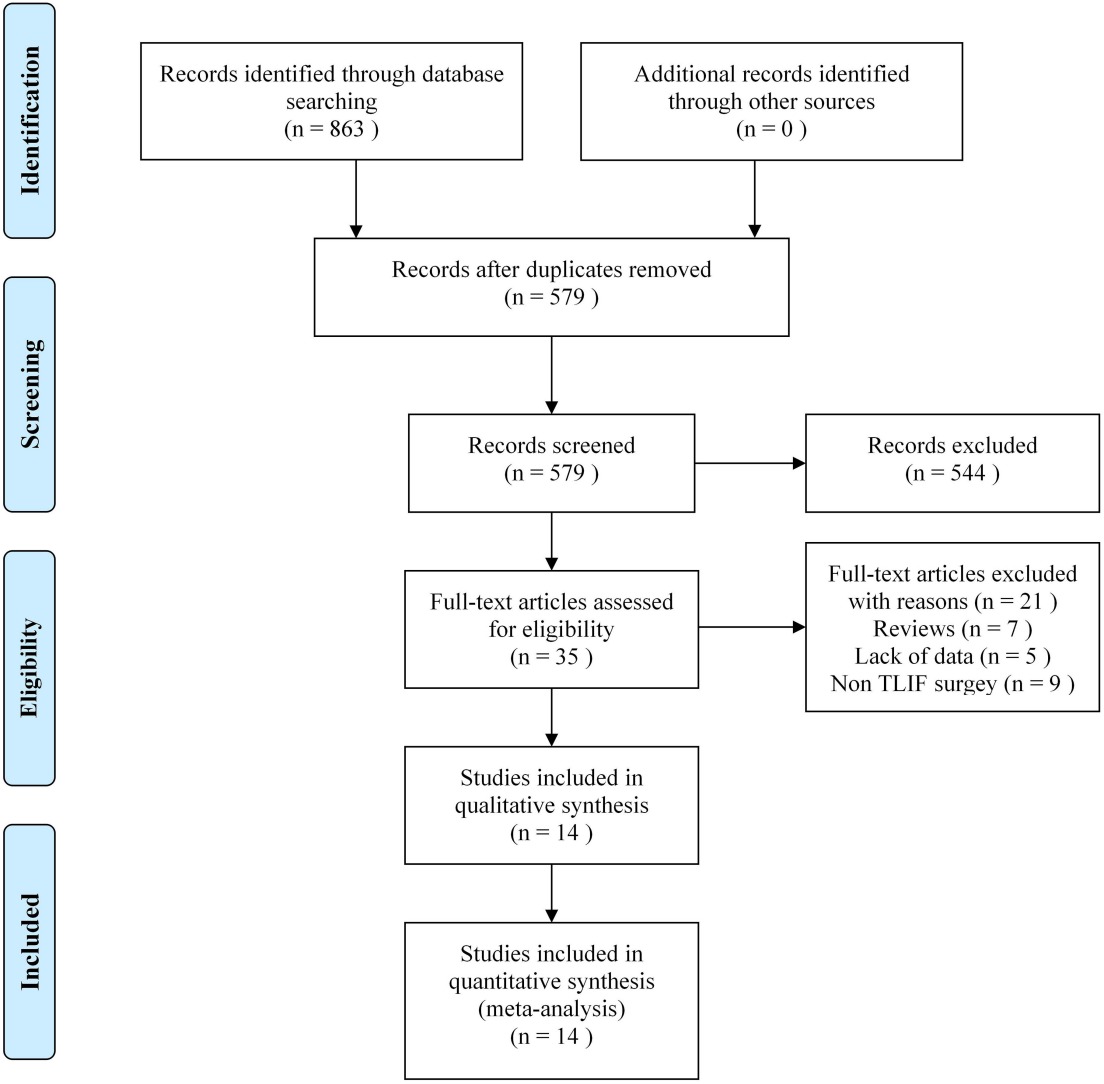
Flow diagram of the study selection process.

Eight hundred sixty-three related literature were retrieved preliminarily. After initial title and abstract screening, 14 studies were assessed for eligibility (20–33), the literature screening process and results were shown in Figure 1. The publication years of the included studies were from 2010 to 2017. The sample size is between 15 and 121. A total of 1086 patients were included, of which 550 patients were in the unilateral group and 536 patients in the bilateral group. Follow-up time was 12–60 months, and the basic characteristics of the included studies were shown in Table 2. The PEDro evaluation of all included studies was high quality (PEDro score ≥ 6). The specific evaluation results were shown in Table 3.

**Table 2:** Basic characteristics of the included studies.

**Study**	**Study**	**Region**	**Sample**	**Age**	**Follow-up**	**Type of**	**Spinal**	**Main**
	**type**		**size (M/F)**	**(years)**	**(months)**	**intervention**	**level**	**outcomes**
			**Unilateral**	**Bilateral**	**Unilateral**	**Bilateral**				
Cheng et al. (20)	RCT	China	23 (11/12)	25 (12/13)	62.3 ± 6.9	64.7 ± 7.5	24	TLIF, capstone cage, autograft	1	VAS, ODI, OP, BL, HT, CR, FR
Conor (21)	Retro	USA	111 (66/45)	40 (26/14)	53.1 ± 11.4	49.2 ± 10.5	24	MIS-TLIF, capstone cage, autograft	1	VAS, ODI, OP, BL, HT
Vigneshwara (22)	Retro	India	112 (64/48)	121 (65/56)	53.6 (26–70)	58.3 (28–80)	24	TLIF, capstone cage, autograft	1	VAS, ODI, OP, BL, HT, CR, FR
Omar (23)	Pro	Egypt	15	15	44.21 ± 1.78	32–55	12	TLIF, PEEK interbody cages, autograft	1	VAS, ODI, OP, BL, HT
Hu et al. (24)	Retro	China	22 (11/11)	23 (10/13)	48.09 ± 10.62	49.78 ± 10.86	38.2 (29–50)	MIS-TLIF, capstone cage, autograft	1	VAS, ODI, JOA, OP, BL, FR
Ren et al. (25)	Retro	China	24 (14/10)	31 (17/14)	64.1 ± 6.3	63.9 ± 6.4	12	MIS-TLIF, capstone cage, autograft	1	VAS, ODI, OP, BL, HT, CR, FR
Jose (26)	Pro	Mexico	33 (17/16)	34 (15/19)	52 ± 16.51	57.38 ± 14.23	12	MIS-TLIF, bullet-nose cage, autograft	1 or 2 or 3	VAS, ODI, SF-36, OP, BL, HT, CR, FR
Liu et al. (27)	Retro	China	22 (8/14)	34 (12/22)	59.16 ± 9.67	58.91 ± 8.51	46.4 (36–60)	TLIF, PEEK cages, autograft	2	VAS, ODI, JOA, OP, BL, HT, CR, FR
Chen et al. (28)	RCT	China	36 (26/10)	42 (29/13)	63	64	24	MIS or open TLIF, cage, autograft	1	VAS, ODI, OP, BL, HT, FR
Gu et al. (29)	Pro	China	35 (17/18)	39 (21/18)	39.0 ± 24.6	42.6 ± 29.1	32	MIS-TLIF, PEEK cage, autograft	2	VAS, ODI, OP, BL, HT, CR, FR
Shen et al. (30)	RCT	China	31 (14/17)	34 (18/16)	57.3 ± 111.7	58.9 ± 10.1	26.6 (18–36)	MIS-TLIF, PEEK cage, autograft	1	VAS, ODI, OP, BL, HT, CR, FR
Zhang et al. (31)	RCT	China	33 (14/19)	35 (10/25)	59.4 ± 10.2	55.7 ± 11.6	24	TLIF, capstone cage, autograft	2	VAS, ODI, SF-36
Nader (32)	Pro	USA	16 (4/12)	20 (6/14)	62.2 ± 13.1	57.3 ± 11.2	12	MIS-TLIF, interbody cage, autograft	1	VAS, ODI, SF-36, FR
Xue et al. (33)	Pro	China	37 (17/20)	43 (18/25)	57.1 ± 8.1	58.2 ± 7.6	18	TLIF, carbon fiber cage, autograft	1 or 2	VAS, ODI, OP, BL, HT, FR

Retro, Retrospective; Pro, Prospective; RCT, Randomized controlled tria; TLIF, Transforaminal lumbar interbody fusion; MIS, minimally invasive; VAS, Visual analog scale; ODI, Oswestry disability index; OP, operation time; BL, Blood loss; HT, Hospital time; CR, Complicayions rate; FR, Fusion rate.

**Table 3:** Assessment of the methodological quality using the PEDro scale.

**Study**	**PEDro scale items** ^*^											**Total score**	**Quality**
	**1**	**2**	**3**	**4**	**5**	**6**	**7**	**8**	**9**	**10**	**11**		
Cheng et al. (20)	Y	Y	Y	Y	Y	N	N	Y	Y	Y	Y	8	Good
Conor (21)	Y	Y	N	Y	Y	N	N	Y	Y	Y	Y	7	Good
Vigneshwara (22)	Y	Y	N	Y	N	N	N	Y	Y	Y	Y	6	Good
Omar (23)	Y	Y	Y	Y	Y	N	N	Y	Y	Y	Y	8	Good
Hu et al. (24)	Y	Y	N	Y	N	N	N	Y	Y	Y	Y	8	Good
Ren et al. (25)	Y	Y	N	Y	N	N	N	Y	Y	Y	Y	6	Good
Jose (26)	Y	Y	Y	Y	Y	N	N	Y	Y	Y	Y	8	Good
Liu et al. (27)	Y	Y	Y	Y	N	N	N	Y	Y	Y	Y	7	Good
Chen et al. (28)	Y	Y	Y	Y	N	N	N	Y	Y	N	Y	6	Good
Gu et al. (29)	Y	Y	Y	Y	Y	N	N	Y	Y	Y	Y	8	Good
Shen et al. (30)	Y	Y	N	Y	Y	N	N	Y	Y	Y	Y	8	Good
Zhang et al. (31)	Y	Y	Y	Y	N	Y	N	Y	Y	Y	Y	8	Good
Nader (32)	Y	Y	Y	Y	Y	N	N	Y	Y	Y	Y	8	Good
Xue et al. (33)	Y	Y	Y	Y	N	N	Y	Y	Y	Y	Y	8	Good

N, no; Y, yes.

* PEDro Scale Items 1, eligibility criteria and source of participants; 2, random allocation; 3, concealed allocation; 4, baseline comparability; 5, blinded subjects; 6, blinded therapists; 7, blind assessors; 8, adequate follow-up; 9, intention-to-treat; 10, between-group comparisons; 11, point estimates and variability.

### Meta-analysis

#### VAS pain

**Figure 2:**
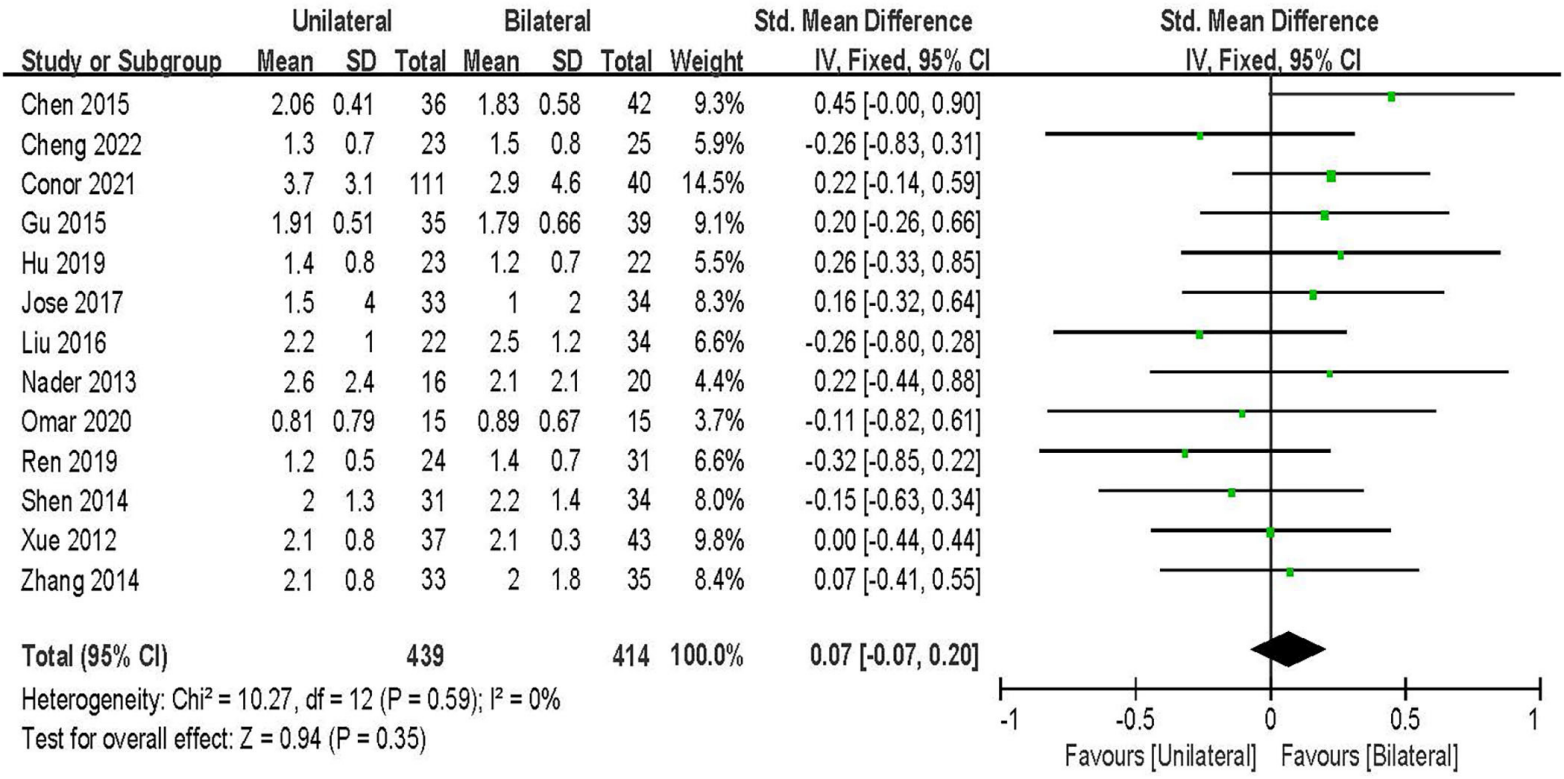
Forest plot of VAS-Low back pain at final follow-up.

VAS-Low back pain score was evaluated in 13 studies including 852 patients (20, 21, 23–33). The results of the fixed-effect model meta-analysis showed that: the overall difference between UPSF and BPSF was not statistically significant [SMD = 0.07, 95% CI (−0.07–0.20), *P* = 0.35; Figure 2].

**Figure 3:**
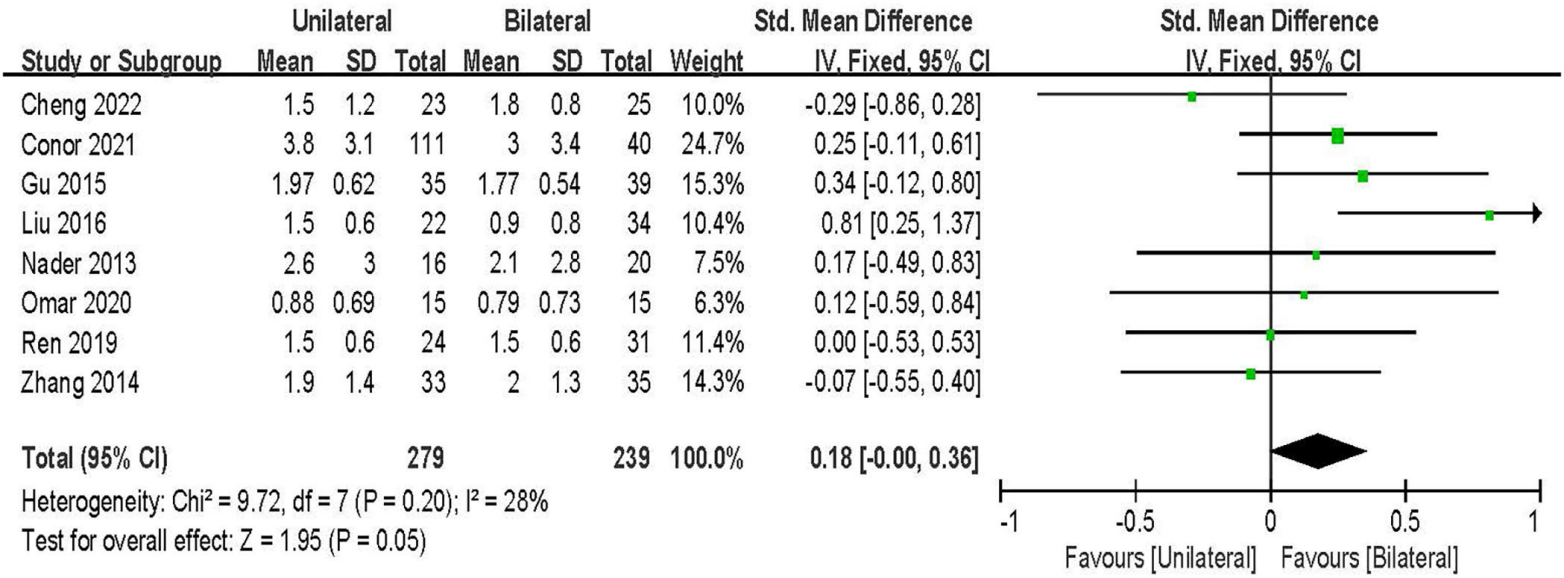
Forest plot of VAS-Leg pain at final follow-up.

VAS-Leg pain score was evaluated in 8 studies including 518 patients (20, 21, 23, 25, 27, 29, 31, 32). The results of the fixed-effect model meta-analysis showed that: the overall difference between UPSF and BPSF was not statistically significant [SMD = 0.18, 95% CI (−0.00–0.36), *P* = 0.05; Figure 3].

#### ODI

**Figure 4:**
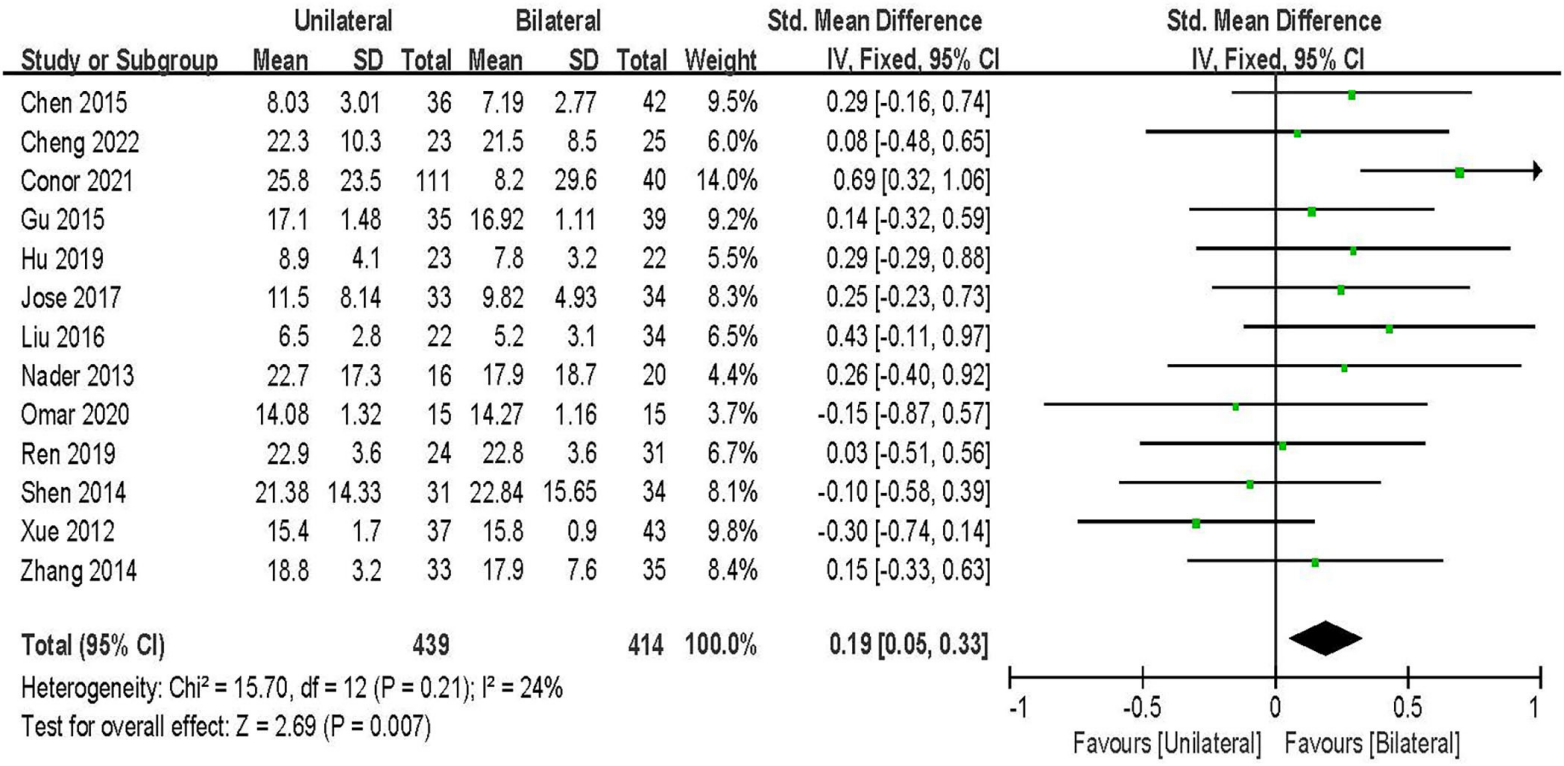
Forest plot of ODI at final follow-up.

ODI score was evaluated in 13 studies including 852 patients (20, 21, 23–33). The results of the fixed-effect model meta-analysis showed that: compared with BPSF, the overall difference between UPSF and BPSF was statistically significant, the ODI score after BPSF was lower, and the degree of lumbar spine dysfunction was less [SMD = 0.19, 95% CI (0.05–0.33), *P* = 0.007; Figure 4].

#### SF-36

**Figure 5:**
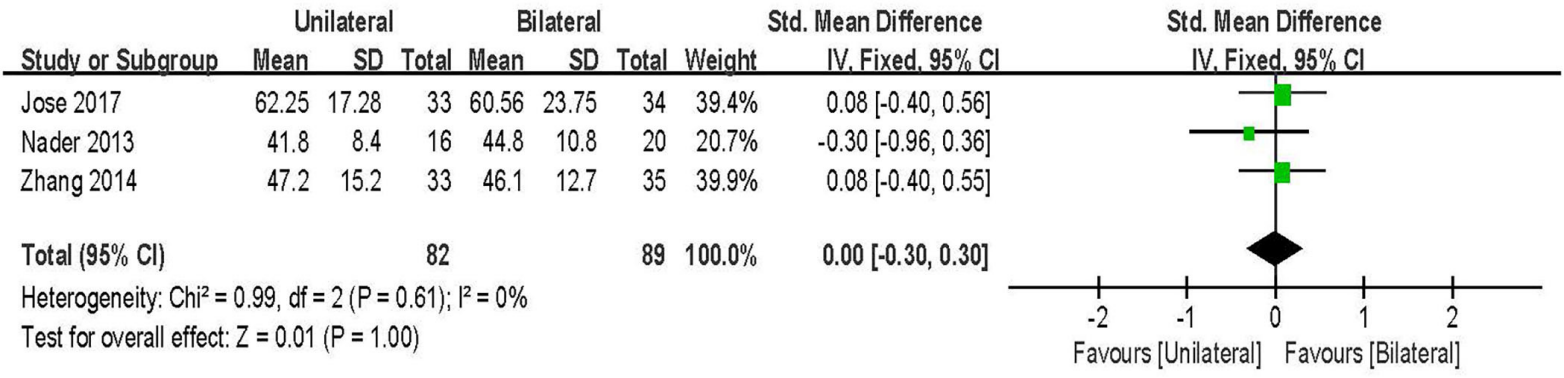
Forest plot of SF-36 at final follow-up.

SF-36 score was evaluated in three studies including 171 patients (26, 30, 32). The results of the fixed-effect model meta-analysis showed that: there was no statistical significance in the overall difference between UPSF and BPSF [SMD = 0.00, 95% CI (−0.30–0.30), *P* = 1.00; Figure 5].

#### Operation time

**Figure 6:**
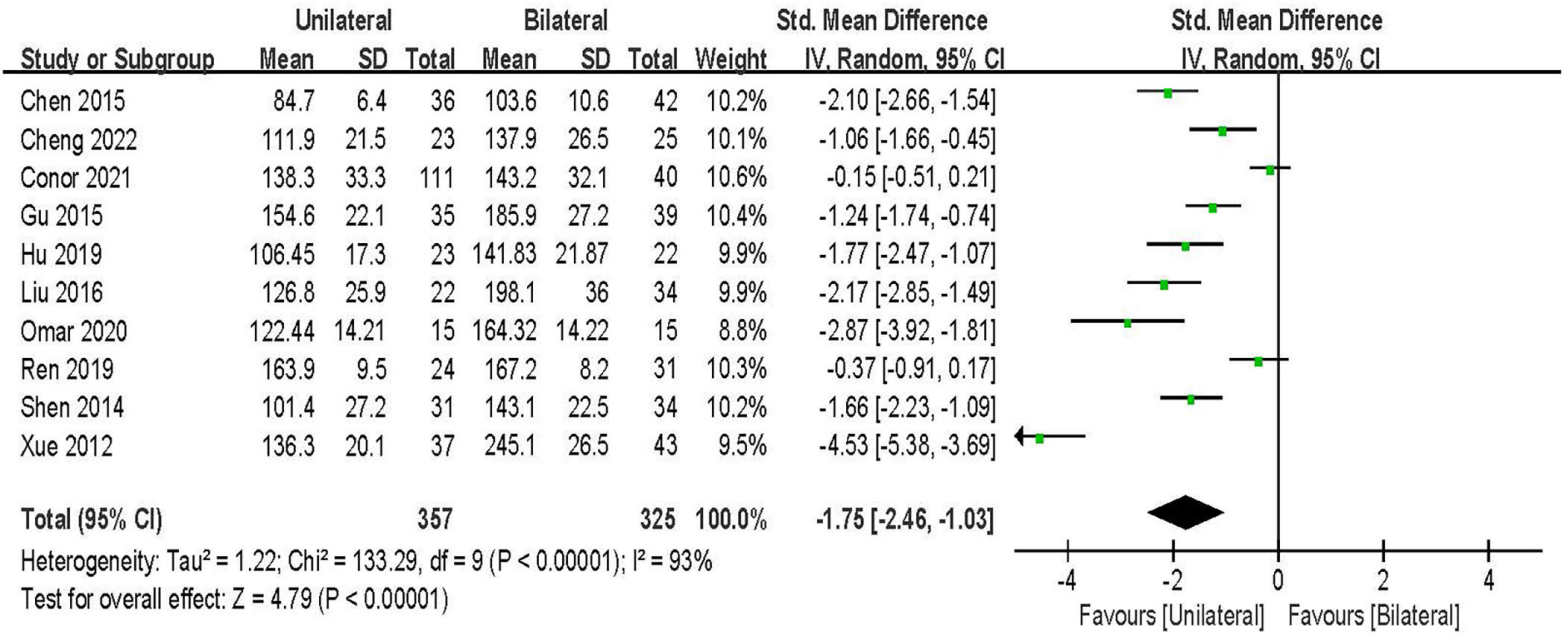
Forest plot of operation time.

Operation time was evaluated in 10 studies including 682 patients (20, 21, 23–25, 27–30, 33). The results of the random-effect model meta-analysis showed that: compared with BPSF, the overall difference between UPSF and BPSF was statistically significant, and the operation time of UPSF was shorter [SMD = −1.75, 95% CI (−2.46 to −1.03), *P* < 0.000 01; Figure 6].

#### Hospital time

**Figure 7:**
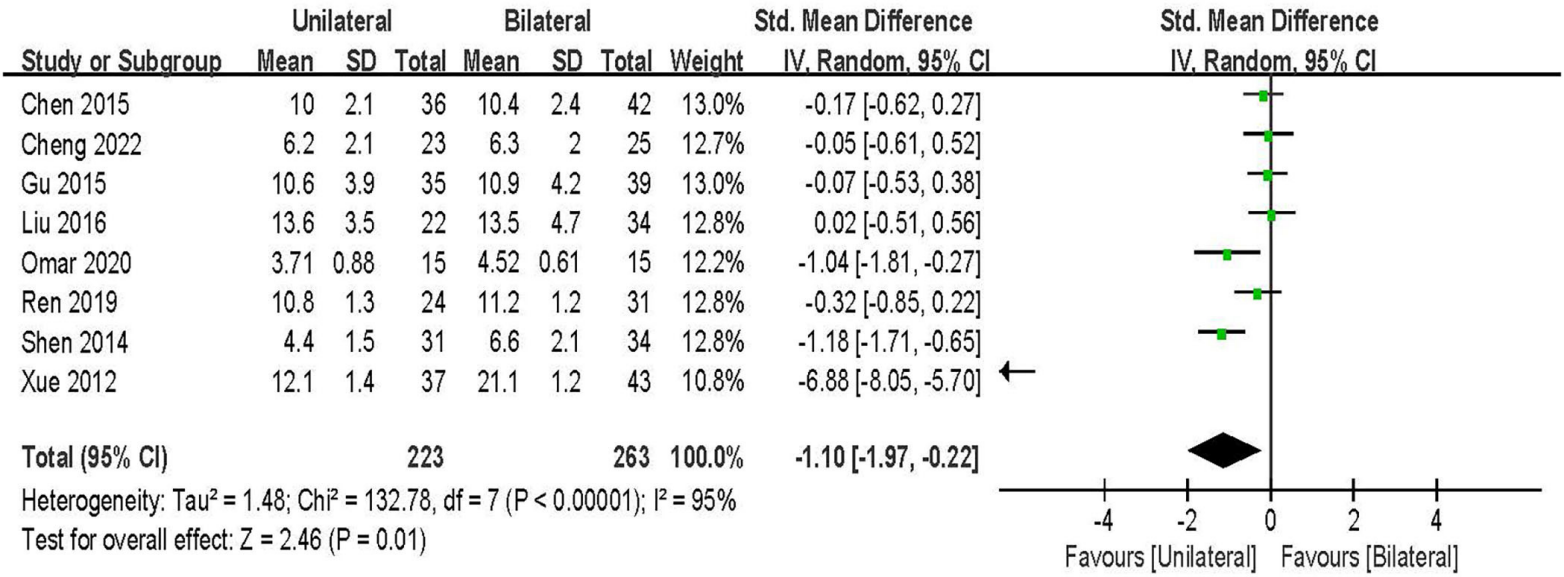
Forest plot of hospital time.

Hospital time was evaluated in 8 studies including 486 patients (20, 23, 25, 27–30, 33). The results of the random-effect model meta-analysis showed that: compared with BPSF, the overall difference between UPSF and BPSF was statistically significant, and the UPSF Hospital time was shorter [SMD = −1.10, 95% CI (−1.97 to −0.22), *P* = 0.01; Figure 7].

#### Blood loss

**Figure 8:**
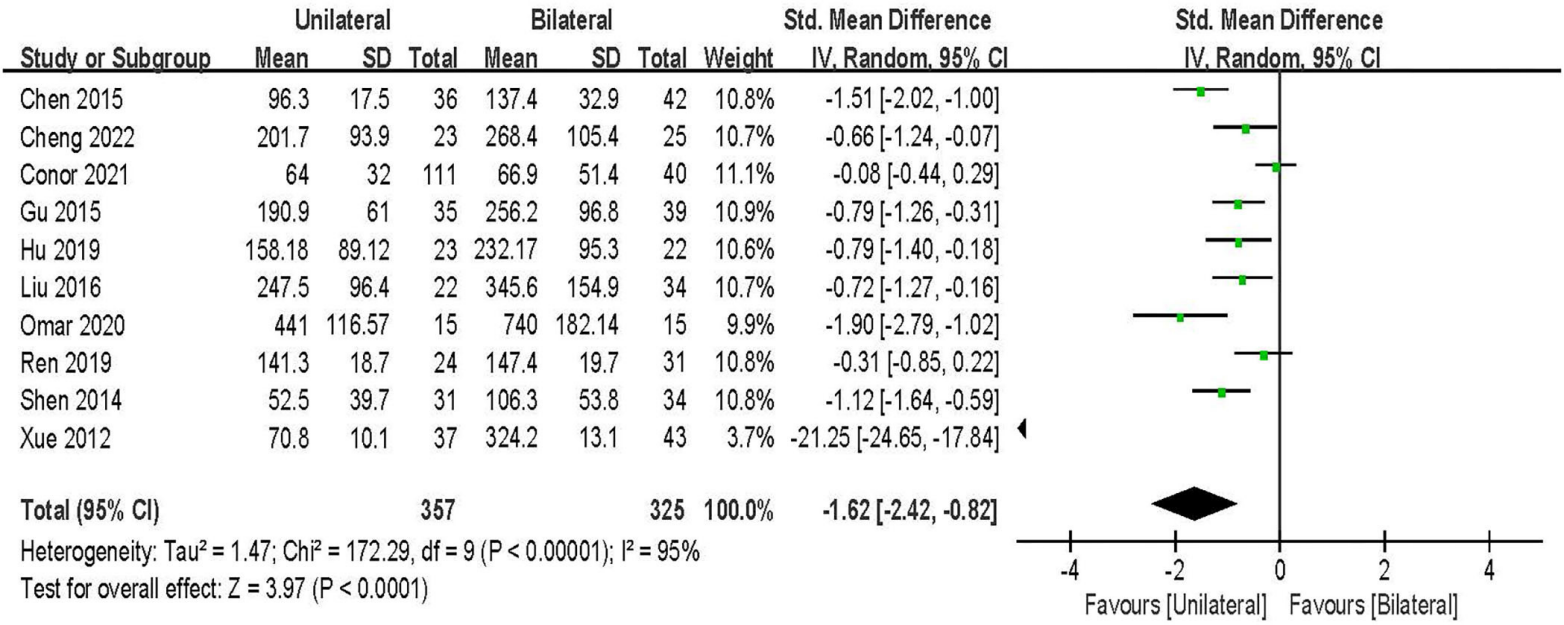
Forest plot of blood loss.

Blood loss was evaluated in 10 studies including 682 patients (20, 21, 23–25, 27–30, 33). The results of the random-effect model meta-analysis showed that: compared with BPSF, the overall difference between UPSF and BPSF was statistically significant, and the blood loss of UPSF was less [SMD = −1.62, 95% CI (−2.42 to −0.82), *P* < 0.000 1; Figure 8].

#### Fusion rate

**Figure 9:**
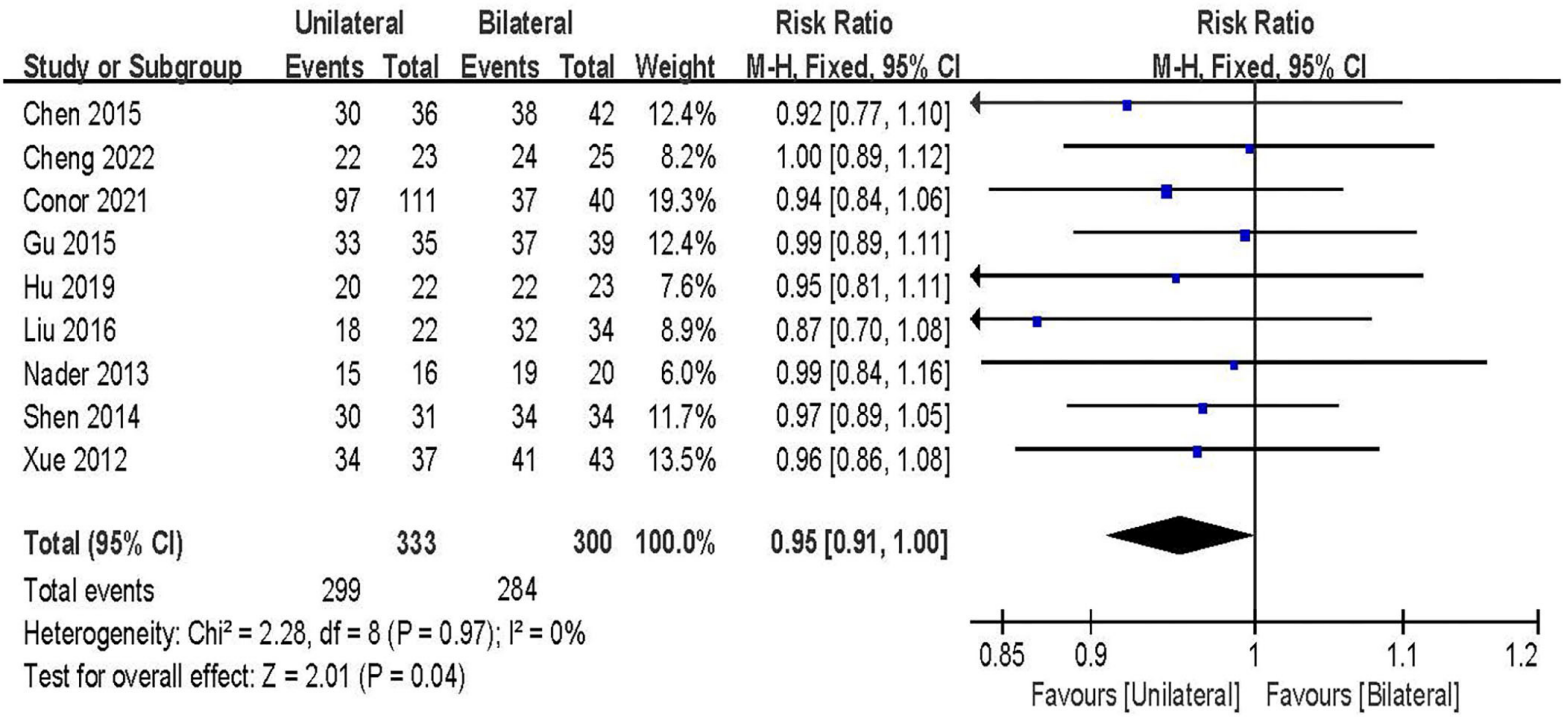
Forest plot of fusion rate.

Fusion rate was evaluated in 9 studies including 633 patients (20, 21, 24, 27–30, 32, 33). The results of the fixed-effect model meta-analysis showed that: compared with BPSF, the overall difference between UPSF and BPSF is statistically significant, the fusion rate after BPSF was higher, and the fusion effect was better [RR = 0.95, 95% CI (0.91 to 1.00), *P* = 0.04; Figure 9].

#### Complications rate

**Figure 10:**
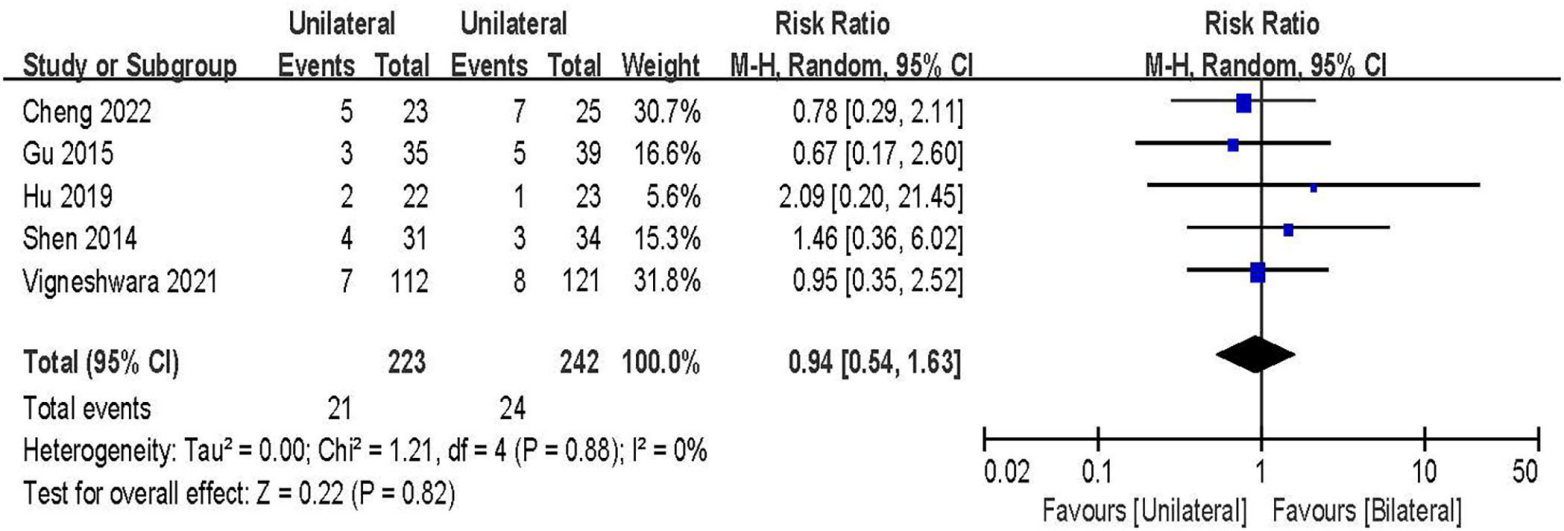
Forest plot of complications rate.

Complications rate was evaluated in 5 studies including 465 patients (20, 22, 24, 28, 30). The results of the fixed-effect model meta-analysis showed that: there was no statistical significance in the overall difference between UPSF and BPSF [RR = 0.94, 95% CI (0.9154–1.63), *P* = 0.82; Figure 10].

#### Operation cost

**Figure 11:**
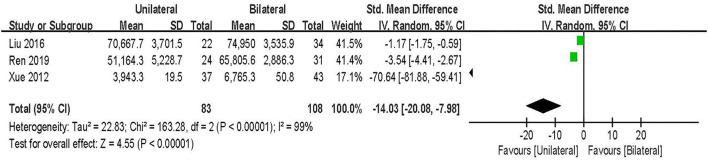
Forest plot of hospital cost.

Operation cost was evaluated in 3 studies including 191 patients (25, 27, 33). The results of the random-effect model meta-analysis showed that: compared with BPSF, the overall difference in Operation cost between UPSF and BPSF was statistically significant, and the UPSFOperation cost was less [SMD = −14.03, 95% CI (−20.08 to −7.98), *P* < 0.000 01; Figure 11].

#### Publication bias

**Figure 12:**
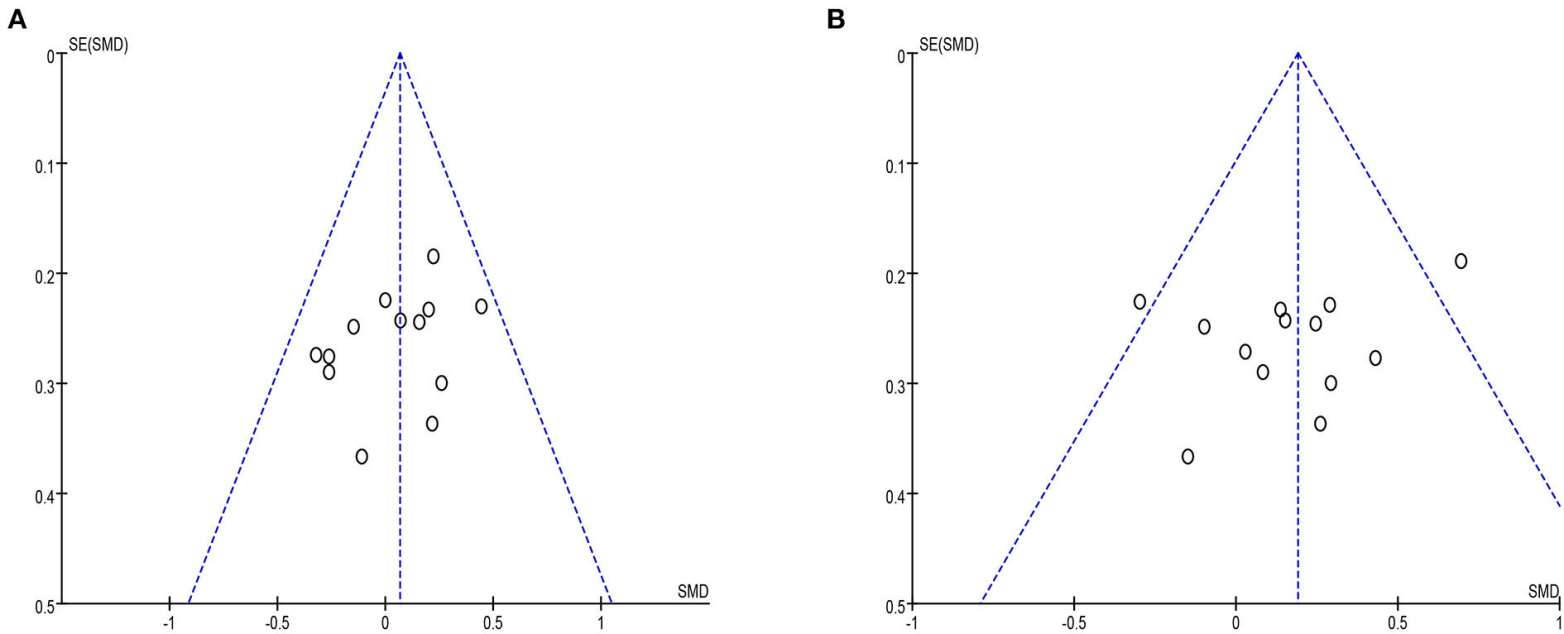
**(A)** The VAS funel plot included in the study. **(B)** The ODI funel plot included in the study.

Funnel plots were drawn for the studies on VAS and ODI with more outcome indicators in the included literature. All VAS studies were distributed within the 95% CI range of the inverted funnel plot, and most of the ODI studies were distributed within the 95% CI range of the inverted funnel plot. The results show that the distribution is vertically symmetrical, indicating that the publication bias is small. As shown in Figures 12A,B.

## Discussion

Spinal fusion surgery has a history of more than 100 years in the treatment of spinal diseases (34). In lumbar fusion surgery, there is little guidance on the various fixation strategies currently used to distinguish the efficacy of different techniques. For LDD such as lumbar disc herniation, spinal stenosis, or spondylolisthesis that fails conservative treatment, lumbar fusion surgery is a classic treatment option. Whether unilateral or bilateral pedicle fixation is required for TLIF surgery has been a topic of debate. In order to understand the latest research trends and related achievements, this meta-analysis systematically reviewed the existing literature on the safety and efficacy of UPSF and BPSF on LDD published in recent years. Update for the latest clinical evidence. The results of this meta-analysis show: (1) Compared with UPSF, the ODI after BPSF was lower, the degree of lumbar spine dysfunction was less, and the fusion rate is higher. (2) Compared with BPSF, UPSF has shorter operation time and hospital time, less blood loss and less operation cost. (3) VAS, SF-36, and complications rate, the difference between the two groups was not statistically significant.

UPSF is less stable than BPSF in axial rotation and lateral buckling, according to research (35). Theoretically, poor biomechanical stability can affect the fusion efficiency of the spine. We included 9 studies that reported the fusion rate, all of which showed that the fusion rate of BPSF was higher. It is hypothesized that after BPSF, the adjacent vertebral bodies can form a more stable connection, which aids in the fusion of the vertebral bodies and thus improves the fusion rate. This modification is consistent with the findings of the previous meta-analysis study (36). The same is true for the changes in ODI results. Although the ODI function of both UPSF and BPSF was significantly improved, after BPSF, the ODI score was lower, and the degree of lumbar spine dysfunction was lower than that of UPSF. Therefore, for the selection of the fixation method, if the above factors are considered, BPSF is more likely to be recommended.

Since the BPSF technique uses a midline approach with significant muscle dissection and retraction on both sides, while UPSF fixation uses a single paramedian fissure approach, even patients with bilateral symptoms can successfully pass through the ipsilateral laminectomy window Perform contralateral decompression, thereby significantly reducing or reducing iatrogenic soft tissue injury (27). Compared with BPSF, operation time and hospital time are shorter, blood loss is less, and surgery cost is less. The reason for consideration is that UPSF only requires exposure of the pedicle on one side and uses a much less invasive approach (31). In addition, the operation time of UPSF is significantly shortened, and the surgical trauma is relatively small, so it is more conducive to the early recovery and rehabilitation of patients after surgery. Therefore, for the selection of the fixation method, if the above factors are considered, BPSF is more likely to be recommended.

The VAS, SF-36, and complications rate of UPSF and BPSF were significantly improved compared with those before surgery. Both methods were effective, but there was no significant difference between the two groups after surgery. In a finite element study of the biomechanical stability of UPSF and BPSF grafts before and after fusion, it was found that graft fusion improved the fixation of the posterior device system. UPSF provides biomechanical stability similar to BPSF in post-fusion level 1 TLIF (37). Currently, the consensus among clinicians is that UPSF should be limited to single-segment posterior lumbar interbody fusions rather than extending to multi-segment fusions due to insufficient fixation strength (32, 38). However, due to the lack of evidence of complications such as cage subsidence and adjacent segment disease, as well as the inherent asymmetry and reduced strength of this system, the use of unilateral instruments may result in disconnection, metal failure, or cage migration (39). Therefore, UPSF cannot be used as an alternative to BPSF for bilevel degenerative spinal diseases. Although BPSF has been considered a standard surgical procedure for spinal fusion surgery to provide rigid lumbar fixation. But the procedure is also suspected of causing degeneration of adjacent segments, device-related osteoporosis, and a higher risk of implant-related complications (40). Considering the clinical pain effect and health survey summary table, and Complications rate, the difference was not significant. Therefore, when we decide to use UPSF or BPSF, other evaluation results after surgery should be considered. In addition, choosing an appropriate surgical approach requires clinicians and patients to explain the advantages and disadvantages, and make surgical choices based on the patient's clinical symptoms.

Xiao et al. concluded in a meta-analysis that UPSF and BPSF have similar clinical outcomes, fusion rates, and complications. However, it is uncertain whether unilateral fixation has the same efficacy and safety profile as bilateral fixation (13). At present, the indications of UPSF are relatively narrow, and it is mainly used for patients with unilateral lower extremity symptoms, no isthmus, and mild degeneration. Unilateral fixation is not suitable for patients with bilateral symptoms or multiple segments (>2). Due to the incomplete anatomy of the non-operative side, unilateral fixation in true lumbar spondylolisthesis cannot achieve sufficient mechanical stability, so UPSF cannot be used (41). From this point of view, BPSF is a better choice. However, the BPSF system can certainly provide a balanced fixation, especially in terms of resistance to axial rotational force and lateral flexion. However, excessively strong internal fixation may lead to stress shielding in the bone graft area, increase the risk of bone graft resorption, osteoporosis, etc., which can affect the bone graft fusion and even increase the incidence of postoperative degeneration of adjacent segments (42). Cannestra et al. found that the expandable interbody cage of UPSF provides greater stability than TLIF using traditional banana-shaped PEEK cages of BPSF (43). Although these studies provide conflicting evidence on whether cage surface area directly affects lumbar spine stiffness, they highlight the effect of increasing surface area on the vertebral endplates to reduce subsidence and thus improve stability. Therefore, optimizing cage placement on the peripheral or posterolateral position on the vertebral endplate to reduce subsidence and endplate damage may result in optimal biomechanical stability (44). In a single-center study of 215 patients with at least 4 years of follow-up, Liu et al. found that UPSF fixation in TLIF could achieve satisfactory clinical outcomes similar to bilateral fixation at medium and long-term follow-up (45). But to avoid cage migration, bullet-shaped cages should not be used in the unilateral group. Since no definite conclusions can be drawn yet, further research is still needed. However, no matter which method is adopted, the selection of the appropriate cage type must be able to open the intervertebral space, the length can more contact the periphery of the endplate, and provide a stable fusion interface that is more conducive to osteogenesis.

To sum up, both UPSF and BPSF interbody fusion can achieve corresponding efficacy and safety in the treatment of LDD. Strictly mastering the surgical indications, anatomical signs and necessary surgical skills is the key to success in choosing which technology. Therefore, unilateral fixation is recommended for lateral lumbar disc herniation with lumbar instability, unilateral lumbar disc root canal stenosis, and lumbar spondylolisthesis with unilateral symptoms (degree I). For patients with severe lumbar instability, severe osteoporosis, lumbar spinal stenosis, or lumbar foramen stenosis who need bilateral decompression or multi-level surgery, more substantial BPSF should be considered.

There are some limitations to this study: (1) Some studies have a small sample size, which may bias the results. (2) There are differences in the basic characteristics of the cases included in the literature, such as the number of fused segments and vertebral body planes, cage differences in surgical methods, and so on, which may affect the surgical effect. (3) Some studies failed to use proper random allocation and concealment methods, which may have resulted in selection bias. (4) Subjective factors influence the evaluation of VAS, ODI, and SF-36. It is relatively large, which may result in some bias. (5) Because all of the included literature is in English and the follow-up time is limited, it is insufficient to detect long-term complications like adjacent segment disease. To obtain conclusive evidence, the sample size must be increased and long-term follow-up is required.

## Conclusions

In conclusion, the available evidence indicates that after UPSF, the amount of bleeding is significantly reduced, the operation time and hospitalization time are significantly reduced, and the blood loss and cost are significantly reduced. The ODI was lower after BPSF, the degree of lumbar dysfunction was lighter, and the fusion rate was significantly improved. The VAS, SF-36 and complications of the two groups were similar, and there was no significant clinical difference. More large-scale, well-designed randomized controlled clinical trials with long-term follow-up are recommended in the future to evaluate the clinical outcomes and long-term impact of these two fixation operations.

## Data availability statement

The raw data supporting the conclusions of this article will be made available by the authors, without undue reservation.

## Author contributions

RZ contributed to the study concept and design, revised, and edited the manuscript. RZ and XX took part in the initial literature search and assessed the eligibilities of feasible studies. JD and RW interpreted the findings and wrote the first draft of the manuscript. RZ, XX, RW, JD, CW, and DL prepared the figures and tables. All authors approved the final version of the manuscript, contributed to the article, and approved the submitted version.

## Funding

This study was supported by the Sports Medicine 2020 Clinical Innovation Project of State General Administration of Sport/Key Laboratory of Sichuan Province (Grant No. LCCX20A02) and Science and Technology Special Project of Sichuan Administration of Traditional Chinese Medicine (Grant No. 2021MS348).

## Conflict of interest

The authors declare that the research was conducted in the absence of any commercial or financial relationships that could be construed as a potential conflict of interest.

## Publisher's note

All claims expressed in this article are solely those of the authors and do not necessarily represent those of their affiliated organizations, or those of the publisher, the editors and the reviewers. Any product that may be evaluated in this article, or claim that may be made by its manufacturer, is not guaranteed or endorsed by the publisher.
